# Therapeutic Levels of Hypothermia Achieved in Isolated Porcine Eyes Using a Scleral Contact Interface

**DOI:** 10.1007/s10439-025-03905-w

**Published:** 2025-11-19

**Authors:** Yukinari Nakamura, Luigi Mecacci, John R. Hetling

**Affiliations:** 1 Richard and Loan Hill Department of Biomedical Engineering, University of Illinois Chicago, 1200 W Harrison St, Chicago, IL 60607 USA; 2 Astellas Pharma Inc., 180 Ozumi, Yaizu, Shizuoka, 425-0072 Japan

**Keywords:** Hypoxia, Hypothermia, Ischemia, Medical device, Neuroprotection, Retina

## Abstract

**Purpose:**

The neuroprotective effect of hypothermia for mitigation of ischemic and hypoxic damage to the retina is well documented, yet technology to achieve targeted, controlled ocular hypothermia *in vivo* is lacking. This study evaluated controlled cooling of ocular tissues using a novel scleral contact eye cooler designed to be practical in a clinical setting.

**Methods:**

Excised fresh adult porcine eyes (*n* = 5) were imaged (at 9.4 T MRI) to document gross anatomy, instrumented with temperature sensors at five key locations, and partially lowered into a warm oil bath (37 °C) to represent surrounding extraocular tissues. A scleral contact ring (SCR) interfaced with an active heat pump was lowered to contact the eye. The SCR was brought to 4 °C and maintained at that temperature using feedback control while monitoring sensor temperatures. After the eye tissues reached thermal equilibrium in the cooled state, the experiment was terminated, and a micro-CT image was obtained to verify the location of each temperature sensor.

**Results:**

Average equilibrium temperatures of the anterior sclera and optic nerve sensors were 10.7 and 30.2 °C, achieved within 3.2 and 11.7 min, respectively. These temperatures have been shown to be neuroprotective against hypoxic damage.

**Conclusion:**

In the non-perfused eye model, therapeutically relevant temperatures could be induced throughout the eye and maintained indefinitely. Demonstration of targeted and controlled cooling of eye tissues using a minimally invasive scleral contact ring will enable *in vivo* therapeutic hypothermia research using a design amenable to clinical translation.

## Introduction

Neuroprotection provided by hypothermia has been extensively studied for ischemic injury related to stroke [1], cardiac arrest [2], traumatic brain injury [3], and perinatal asphyxia [4]. Systemic cooling in a clinical setting is achieved using surface cooling or endovascular cooling and comes with significant associated risks [5, 6]. More targeted cooling of brain tissue has been demonstrated using contact cooling of only the head [7, 8], transnasal evaporative cooling [9] and circulation of cold saline through the pharyngeal region [10]. Evidence for a beneficial effect has led to clinical adoption of hypothermia treatments in these contexts that are supported by commercially available cooling systems.

Retinal ischemia and the subsequent hypoxia can lead to retinal cell death and associated vision loss within tens of minutes; hypothermia as a neuroprotective treatment has been studied in this context as well [11]. Tissue temperatures of 28 to 35 °C, known as mild or moderate hypothermia, have shown protective effects against ischemic damage [11]. Research focusing on the benefits of ocular hypothermia has most commonly employed retinal cell cultures or explants [12, 13]. While these studies have been crucial in understanding the effects and underlying mechanisms of therapeutic hypothermia in the eye, it will ultimately be necessary to conduct safety and efficacy studies in relevant *in vivo* animal models and, eventually, human subjects using a practical method to achieve controlled cooling of the eye. To date, *in vivo* studies of the benefits of hypothermia in the eye have used three basic approaches: intraocular irrigation, extraocular irrigation, or whole-body cooling. None of these approaches are amenable to practical translation for application in a clinical setting.

*In vivo* intraocular irrigation has been studied primarily in the context of surgical vitrectomy [14–16]; this invasive approach is not an attractive option for clinical treatment of retinal ischemia. Extraocular irrigation has been employed in rats and compared to whole-body cooling in the same study [17]; irrigation with 13 °C gel resulted in a temperature of 32 °C measured just behind the ocular globe. *In vivo* cooling has most often been employed in mice or rats either by placing awake animals in a cold environment [18–21] or by using ice packs with anesthetized animals [8, 22].

Studies using large animal models (porcine, bovine) have primarily applied hypothermic treatments to *ex vivo* retinal preparations [1, 23, 24]. One recent study used direct convective cooling of the pre-chiasmic optic nerve in a goat requiring surgical access to the target tissue [25]. The application of a copper cooling plate to the eyelid (closed eye) has been proposed for human use, but no measurements of cooling effectiveness were reported [26].

Despite extensive evidence of neuroprotection in the retina and optic nerve associated with hypothermic treatment, no commercially available systems, other than topical ice packs, are available for cooling the eye in a targeted manner. Systemic cooling could be used but comes with known risks and requires bulky, time-consuming equipment and procedures [5, 6].

Gongal et al. [27] used a computational model of heat flow in the non-perfused eye to explore cooling of the neural retina using an active heat sink that contacts only the accessible area of the eye surface surrounding the cornea (but not the cornea itself), representing a minimally invasive, clinically feasible approach. The study simulations predicted therapeutic temperatures could be reached across the entire retina within ten minutes using this approach. The primary aim of the present study was to empirically validate the approach described in Gongal et al. [27] in isolated porcine eyes. A preliminary version of this work has been reported [28].

## Materials and Methods

### General Protocol

**Fig. 1:**
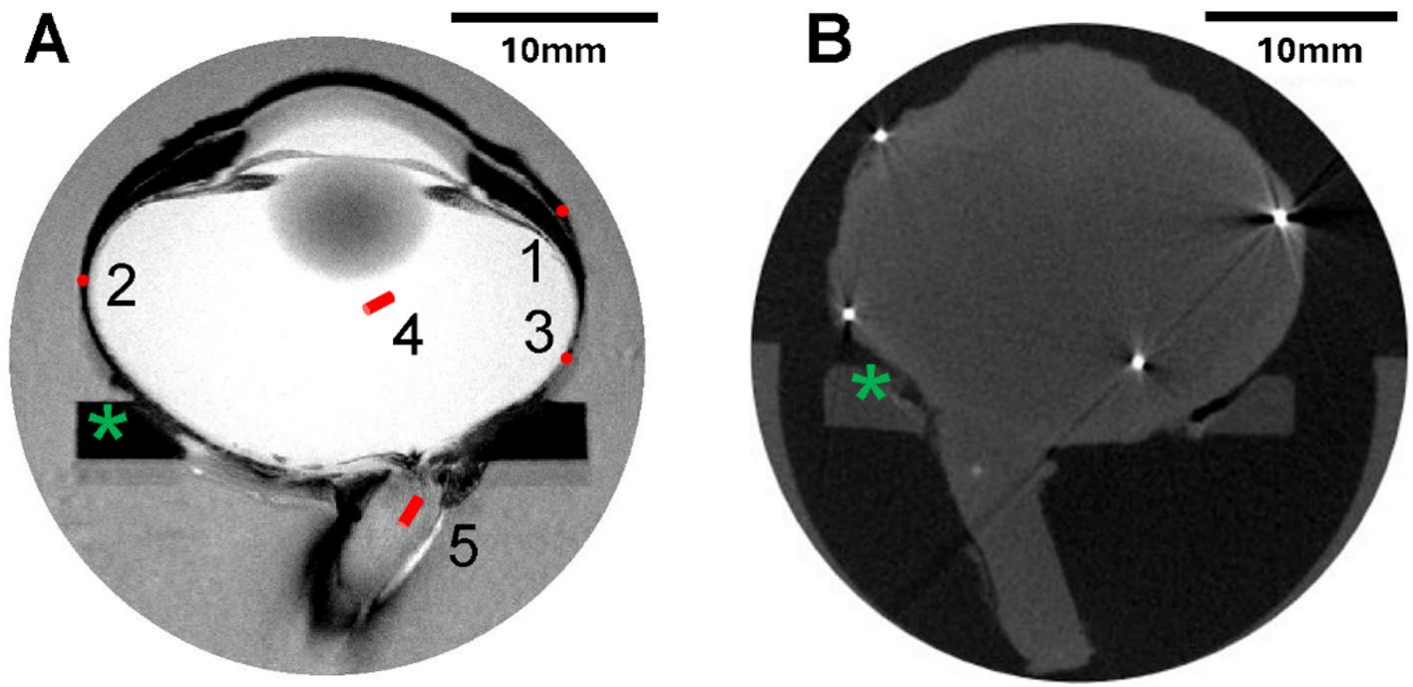
Representative images of porcine eyes used to determine the locations of implanted temperature sensors. **A** MRI slice (horizontal plane, 0.078 × 0.078 × 0.800 mm voxel size) at the approximate midline of the eye globe. The target positions and orientations for the five implanted temperature sensors are indicated by red cylinders (approximately to scale) and numbered: 1, anterior sclera; 2, equatorial sclera; 3, posterior sclera; 4, vitreous; 5, optic nerve. **B** Micro-CT slice (54*.*6 µm voxel size) at the approximate midline of the eye globe. Four of the five temperature sensors appear as bright white spots in this image slice. The optic nerve sensor is slightly out of the image slice and appears as a light gray spot. In both panels, the green asterisks indicate the acrylic eye support used throughout the imaging and data collection phases of the experiment

**Fig. 2:**
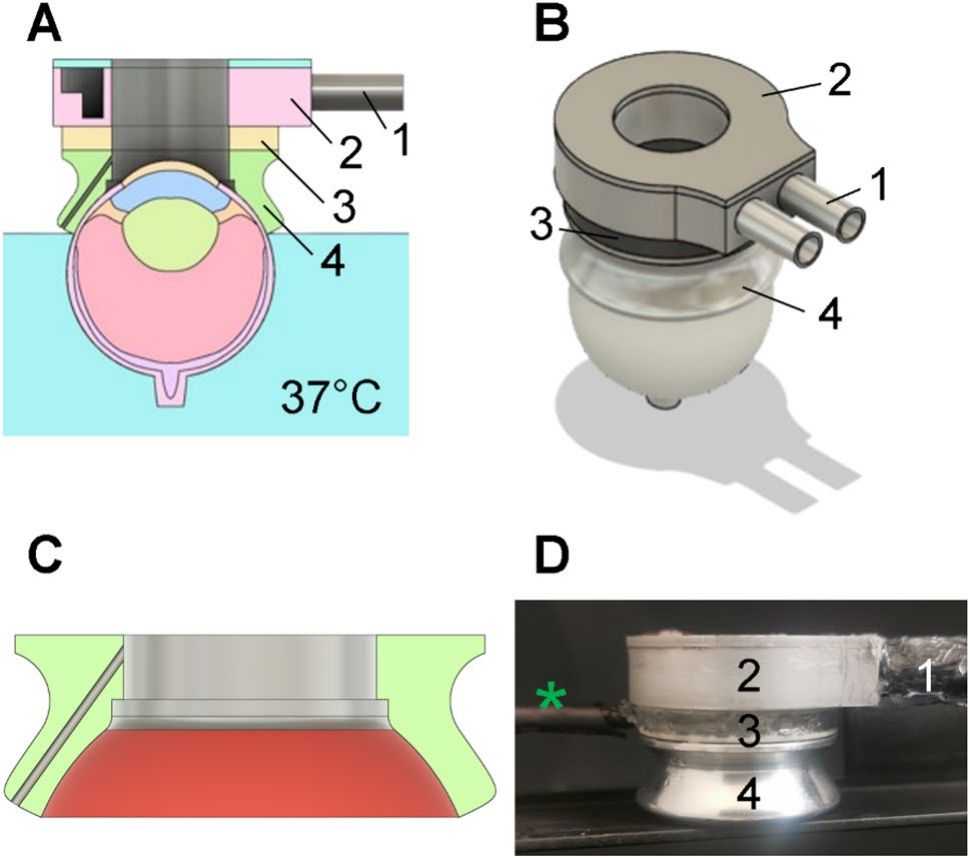
Components of the scleral contact eye cooler (SCEC). **A** Cross-section of CAD model of the SCEC in position on a schematic representation of a porcine eye. Main components identified by number: 1, cooling fluid inlet/outlet; 2, heat exchanger; 3, thermoelectric heat pump (TEC); 4, scleral contact ring (SCR). The warm oil level is shown in light blue. **B** Solid model of the SCEC and eye; component numbers are the same as in panel **A**. **C** Detail of the SCR (cut away) with the intended scleral contact area highlighted in red. **D** Photograph of an assembled SCEC; component numbers are as in panel **A**. Green asterisk indicates the power supply wires for the TEC (not shown in panels **A** and **B**). The junction with the fluid inlet/outlet tubes was coated with silicone sealant in this assembly. The total height of the prototype SCEC was 23 mm

**Fig. 3:**
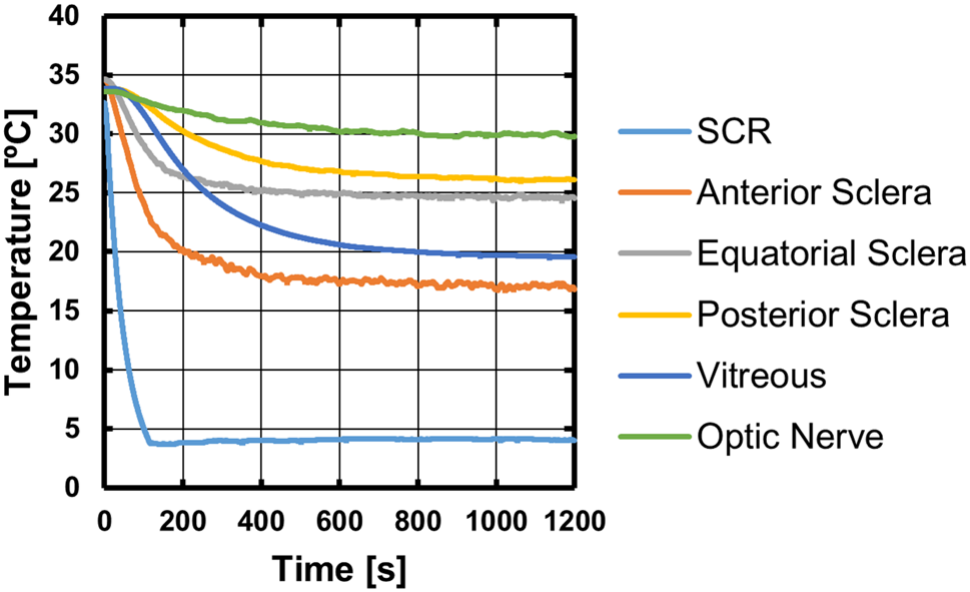
Temperature vs*.* time curves for one eye. Each trace plots the temperatures observed at one of the six sensors, as indicated in the color legend. Trace SCR is for the feedback sensor embedded in the scleral contact ring. Positions of the other sensors are as shown in Fig. 1A. The SCR set temperature was set to 4 °C at *t* = 0 and remained at that value for 20 min

Fresh porcine eyes were imaged using MRI to document gross anatomy. Eyes were then instrumented with temperature sensors implanted at five key locations (Fig. 1). Mounted on a support ring with the cornea facing upward, the instrumented eye was lowered into a warm oil bath (37 °C) to represent the extraocular tissues, and a scleral contact ring (SCR, held at a typical corneal surface temperature, 34.5 °C) was brought into contact with the anterior sclera of the eye (Fig. 2). Once the eye tissues reached thermal equilibrium (approximately 30 min), power to the SCR was turned off and after a few additional minutes, temperatures at all sensors were recorded as the starting temperatures for the experiment. The set temperature of the SCR was then changed to 4 °C. Temperatures at the five sensor locations were monitored for 20 min, long enough to reach thermal equilibrium in the cooled state at each location (Fig. 3). Following the experiment, the sensor wires were cut close to the eye surface, and the eye was imaged using micro-CT to document the location of each sensor (Fig. 1).

### Eye Preparation

Fresh porcine eyes (Animal Biotech Industries, Inc., Doylestown, PA) were harvested with at least 13 mm of optic nerve attached, packed in cold saline, and shipped overnight. Excess extraocular tissue was carefully removed, and each eye was mounted in a custom acrylic support ring using a few drops of cyanoacrylate adhesive. Under an ophthalmic surgical microscope, five locations on the eye were identified using anatomical landmarks and marked with a fine-point permanent marker. At each location, an incision was created using the tip of an 18 Ga needle, and a small cylindrical temperature sensor (3.3 mm long × 0.5 mm diameter NTC thermistor, 200 ms response time, part number GA10K3MCD1, TE Connectivity Ltd., China) was inserted to a predefined depth (Fig. 1A). Sensors in scleral tissue (locations 1, 2 and 3 in Fig. 1A) were oriented parallel to the scleral surface. The sensor in the vitreous humor (location 4 in Fig. 1A) was approximately in the center of the globe of the eye, though the precise position was difficult to control. The sensor in the proximal optic nerve (location 5 in Fig. 1A) was oriented parallel to the axis of the nerve stump. A drop of cyanoacrylate adhesive was applied to each incision site to secure the sensor wires in place. Precise locations of all sensors were documented using CT imaging after each experiment (Fig. 1B).

### Scleral Eye Contact Cooler

The scleral contact eye cooler (SCEC) is comprised of three main components (Fig. 2). The scleral contact ring (SCR, component 4 in Fig. 2) was custom machined of aluminum with one side shaped to conform to the anterior scleral surface of the eye, with an intended scleral contact area of 390 mm^2^ (the curved surface highlighted in red in Fig. 2C). The cornea remained exposed to the ambient air through a 14 mm diameter void in the center of the SCEC. One temperature sensor was embedded within the SCR and used to monitor the SCR temperature and provide feedback to the SCEC temperature control circuit.

Heat was actively drawn from the SCR by an annular thermoelectric heat pump (TEC, component 2 in Fig. 2; model number RH14-14-10-L1-W4.5, Laird Thermal Systems, Durham, NC). Heat was in turn drawn from the TEC *via* a custom-machined aluminum heat exchanger (component 2 in Fig. 2). Cooled fluid (50:50 solution of polyethylene glycol and distilled water) was continuously circulated through the heat exchanger at a rate that was sufficient to allow the TEC to function over a wide range of ambient temperatures (approximately 150 mL/min). The TEC-SCR and TEC-heat exchanger interfaces were bonded with thermally conductive epoxy (model OB-101-2, Omega Engineering, Inc., USA). The TEC was operated in a current-controlled manner, at a maximum power of 8.6 W, with feedback from the SCR sensor and a control circuit (a simple comparator with hysteresis, ∆ ≈ 0.7 °C) which allowed the SCEC to reach and maintain target temperatures within the range of 0–37 °C.

### In Vitro Model System

Instrumented eyes, with the cornea facing up, were submerged to a level approximately 4 mm below the limbus (just below the SCEC) in a gently circulating canola oil bath held at 37.0 °C to simulate the extraocular tissues (Fig. 2A). Temperature sensor wires were routed to a quick-connect interface for the data acquisition system. The SCEC was lowered *via* a linear translator onto the eye until no light (from a focal source temporarily placed within the central void of the SCEC) could pass between the SCEC and the eye in any direction. Intraocular pressure was maintained at 15.2 mmHg by using a 27 Ga needle, inserted through the cornea into the anterior chamber, connected to a bag of room-temperature phosphate-buffered saline (PBS) suspended 21 cm above the level of the eye [29]. Flow of PBS into the eye was negligible and expected to have a negligible effect on ocular temperature. After temperature vs. time data were obtained, the needle was removed from the cornea and the hole sealed with cyanoacrylate prior to micro-CT imaging. The ambient temperature and relative humidity in the lab room during data recording were approximately 23–25 °C and 55–60%, respectively.

### Data Acquisition

A custom six-channel signal conditioning circuit was built to convert the changes in temperature sensor resistances into linear changes in voltage prior to digitization at a sampling rate of 10 Hz (model DI-2108, DataQ Instruments, Akron, OH, USA). Because new temperature sensors were used for each eye, every experiment began with calibrating all six sensor channels to a sensitivity of 0.48 V/°C (± 0.05 °C) within the range of 0–37 °C using a dry block temperature calibrator (Fluke 9102S-156) with a custom-machined insert.

### Imaging

Magnetic resonance imaging (MRI) was used to document the gross anatomy of each eye prior to implanting the temperature sensors (9.4T small bore magnet, Agilent, Santa Clara, CA); Fig. 1A shows a representative cross section (36 slices obtained for each eye). MRI scans were acquired with 0.078 × 0.078 × 0.8 mm voxels, repetition time (*T*_R_) = 550.0 ms, time to echo (*T*_E_) = 7.50 ms, and flip angle = 20°. After each experiment, the sensor wires were cut close to the eye surface and the eye was imaged using microcomputed tomography (micro-CT, X3000 Industrial CT Inspection System, NSI, Rogers, MN) to document the precise location of each of the five sensors (voxel size 54*.*6 µm); image data were reconstructed using efX-CT software. A representative CT image is shown in Fig. 1B. All imaging was performed with the eyes mounted in a custom oil-filled chamber to maintain a consistent position and orientation in the imaging chambers, to prevent shape changes of the eye due to drying, and to provide high, artifact-free contrast at the eye boundaries.

Micro-CT images were converted to DICOM stacks and imported into 3D Slicer (open access) [30]. Within 3D Slicer, the MRI and micro-CT images were registered using rigid landmark registration to reveal the sensor positions within the eye. Eyes number 2–5 were registered to the image of eye number 1 for comparison of sensor positions across eyes. After the registration process, the locations of each sensor were determined relative to the mean position (across eyes) of each sensor. The mean position across eyes is referred to as the target location below (Table 1 and related text).

**Table 1:** Summary of sensor positions derived from three-dimensional micro-CT scans

	Distance from average position (mm)				
	Anterior sclera	Equatorial sclera	Posterior sclera	Vitreous	Optic nerve
Eye 1	2.2	3.0	1.4	4.8	2.7
Eye 2	2.4	0.5	1.0	5.3	3.3
Eye 3	1.8	1.3	1.8	2.6	1.0
Eye 4	1.8	3.3	1.5	4.1	1.2
Eye 5	0.8	2.2	1.3	4.4	1.8
Mean (SD)	1.8 (0.6)	2.1 (1.2)	1.4 (0.3)	4.2 (1.0)	2.0 (1.0)

The top five rows contain the distance of that sensor (column) for that eye (row) from the mean position of that sensor across eyes. Average distances across eyes for that eye (row) from the mean position of that sensor across eyes. Average distances across eyes for each sensor position are given in the sixth row, with the corresponding standard deviation in parenthesis

## Results

Each experiment yielded six temperature *vs* time curves, one for each temperature sensor. Results from one experiment are plotted in Fig. 3. After a holding period with the set temperature of the SCR at 34.5 °C, the set temperature was changed to 4 °C for eye cooling at time zero. The former temperature was taken as the typical temperature of the exposed anterior surface of the eye *in vivo* [31]. The latter temperature was chosen as a minimum safe temperature that would both provide maximum cooling of deeper eye tissues and avoid the risk of hypothermic damage to the tissues in immediate contact with the SCR. 4 °C is a temperature commonly employed for the preservation of donor tissues prior to transplant [32] and has been used for deep hypothermic treatment of the optic nerve [25].

As can be seen in Fig. 3, the scleral contact ring (SCR) transitioned from the initial holding temperature near 34.5 °C to 4 °C in approximately 2 min. The temperatures observed at each sensor location within the eye transitioned at varying rates from their starting temperatures to distinct final equilibrium temperatures. For the sensors implanted in scleral positions and in the optic nerve, the final equilibrium temperature and the transition rate between initial and final temperatures were correlated with distance between the sensor and the SCR contact area (Fig. 1A). The sensor in the vitreous was farther from the SCR contact area than the equatorial scleral sensor, but experienced a more rapid temperature transition, and reached a lower final temperature; this is likely due to the proximity of the scleral sensor to the warm oil bath surrounding the posterior of the eye. Importantly, the optic nerve sensor experienced a temperature reduction of nearly 5 °C.

An attempt was made to achieve consistent positioning of the temperature sensors in each eye. The final position was verified with micro-CT imaging. The mean sensor position achieved for each location shown in Fig. 1A, across the five eyes, was determined and taken to be the target position.

Table 1 summarizes the distances (Euclidean, straight-line distance in three-dimensional space) between each individual sensor and the target position for that sensor. Scleral and optic nerve sensors were, on average, 1*.*8 ± 0.8 mm (mean ± one standard deviation) from the target locations. For the vitreous target location, sensor position was more variable, with distances across the five eyes of 4*.*2 ± 1.0 mm (mean ± one standard deviation). Given the reasonable consistency of sensor position across eyes, direct comparison of repeated measures across eyes was justified.

**Fig. 4:**
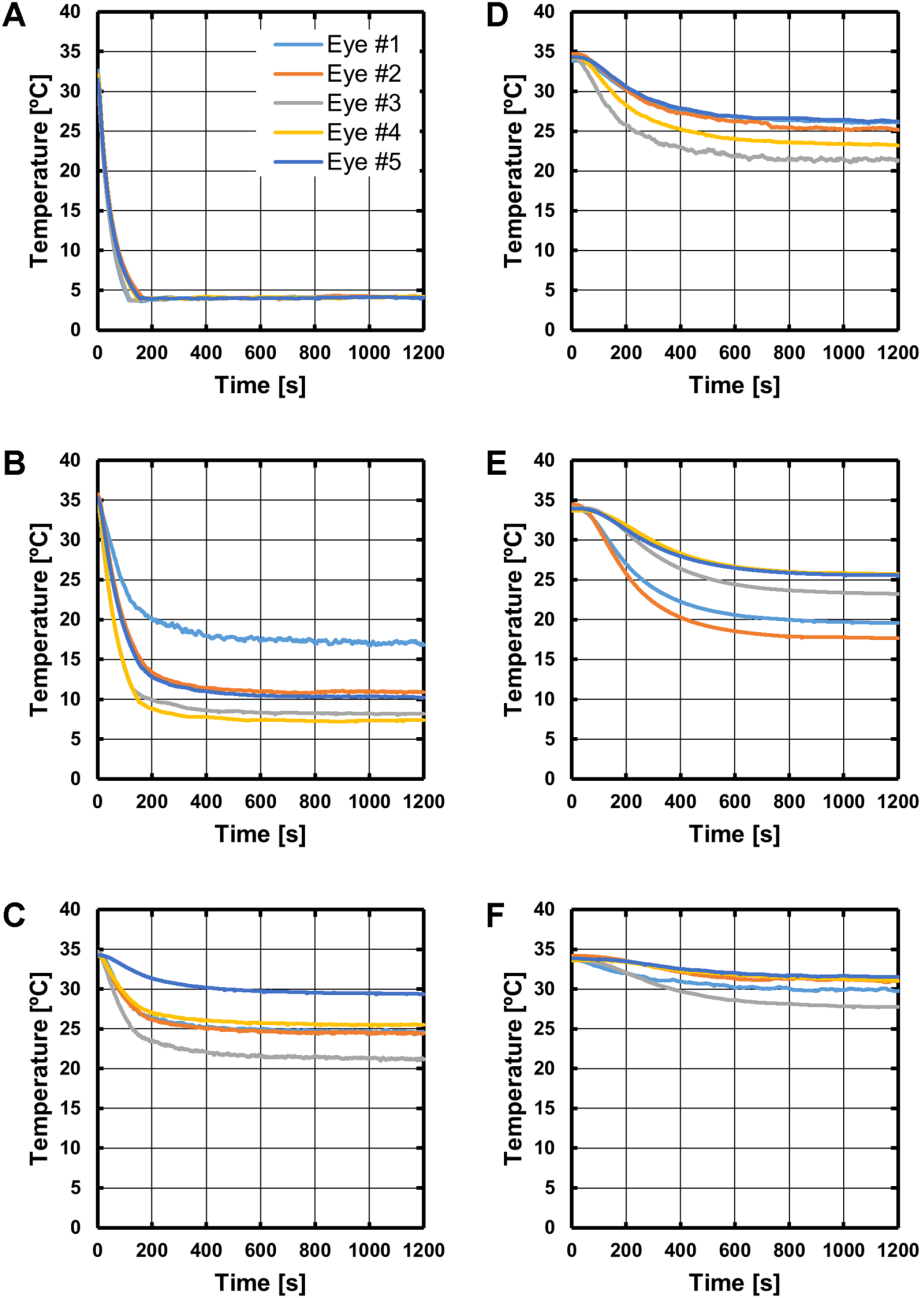
Temperature vs*.* time curves for all five eyes, grouped by sensor position. In all panels, the SCR set temperature was changed to 4 °C at *t* = 0 and remained at that value for 20 min. Each trace color indicates data from one eye across the panels. Panels show data from the sensors located at **A** scleral contact ring (SCR, feedback sensor), **B** anterior sclera, **C** equatorial sclera, **D** posterior sclera, **E** vitreous, and **F** optic nerve as shown in Fig. 1A

Figure 4 summarizes the temperature data across all five eyes, where each plot contains the five temperature vs*.* time curves for one target sensor location. The curves are generally similar within each plot. To quantify repeatability across eyes, a root mean squared error (RMSE) value was calculated for each curve, where the error was defined as the difference between that curve and the average curve across eyes. The minimum, maximum and average RMSE values at each sensor position are summarized in Table 2. The RMSE values are in units of degrees C and should be considered in the context of the final change in temperature experienced at each sensor location (at *t* = 1200 s). The RMSE values were therefore converted to a percentage of the mean total temperature change observed at each target sensor location (Eq. 1); the average RMSE% value ranged from 11 to 29%. The observed variability in measured temperature values across eyes is most likely due to the variability in sensor placement; for each target sensor location, the achieved sensor location could be 2–6 mm from the mean location across eyes (**Max** row in Table 1).

**Table 2:** Repeatability of SCEC-induced temperature changes across eyes

	SCR	Anterior sclera	Equatorial sclera	Posterior sclera	Vitreous	Optic nerve
Min	0.2	0.5	0.4	1.0	1.1	0.6
Max	0.4	6.4	4.2	3.1	4.4	2.0
Average	0.3	2.8	1.8	1.6	2.8	1.0
RMSE (%)	1.1	11.3	19.4	16.6	23.9	28.8

Root mean squared error (RMSE) values were calculated between temperature vs*.* time curves for individual eyes and the average curve for the five eyes, for each sensor location. All values in the top three rows are in degrees C. The average error across eyes (row 3, Average) was converted to a percentage of the average total temperature excursion observed at that sensor location (row 4, RMSE%) using (1)

1 $$\mathrm{RMSE\%}= \frac{\mathrm{RMSE}}{{T}_{\mathrm{Initial}}-{T}_{\mathrm{Final}}}\times 100.$$

**Fig. 5:**
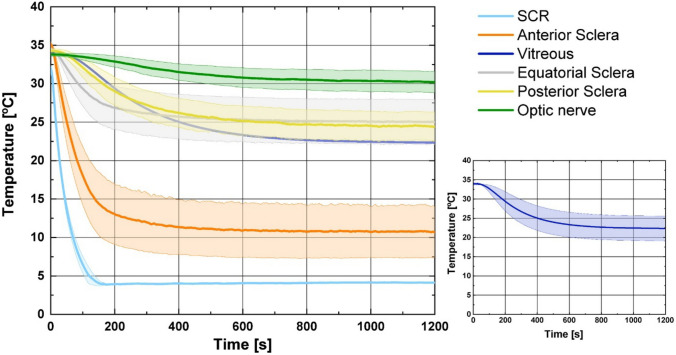
Temperature vs*.* time curves averaged across the five eyes. Each bold trace in the main panel plots the mean temperatures observed at one of the six sensor locations, as indicated in the legend. The shaded area represents ± one standard deviation, at each time point, for the specific location. For clarity, the shading is not shown for the Vitreous trace in the main panel, but is shown in the smaller plot at the bottom right. Trace SCR is for the feedback sensor embedded in the scleral contact ring. Target positions of the other sensors are shown in Fig. 1A. The SCEC set temperature was changed to 4 °C at *t* = 0 and remained at that value for 20 min in all experiments

Given the reasonable consistency in sensor position (Table 1) and the general repeatability of observed temperature change across the five eyes (Table 2), the data from the five eyes were averaged and are plotted in Fig. 5. The curves in Fig. 5 can be taken as reasonable predictions of temperature change at each sensor location when the SCR is held at 4 °C. These averaged curves were analyzed for the time taken to reach 50% and 90% of final temperature at each sensor location; these values are summarized in the bottom two rows of Table 3. Final temperatures shown to be therapeutically relevant in studies of ischemic damage were reached at every sensor location. The anterior sclera, closest to the SCR, reached final equilibrium temperature in 3.2 min, on average. The optic nerve stump, farthest from the SCR, required nearly twelve minutes to reach a final equilibrium temperature that was, on average, 3.6 °C below the starting temperature.

**Table 3:** Summary of initial and final temperatures for each sensor location and time taken to reach 50% and 90% of the final temperature change (*t*_50_ and *t*_90_)

	SCR	Anterior sclera	Equatorial sclera	Posterior sclera	Vitreous	Optic nerve
Initial (°C)	32.1	35.1	34.5	34.3	34.0	33.8
50% (°C)	18.1	22.9	29.8	29.3	28.2	32.0
90% (°C)	6.9	13.1	26.0	25.4	23.5	30.6
Final (°C)	4.1	10.7	25.0	24.4	22.3	30.2
*t*_50_ (s)	33.4	62.6	91.0	186.6	241.2	314.1
*t*_90_ (s)	95.2	194.2	337.8	531.7	569.0	703.1

The temperatures at 50% and 90% of the total excursion (Initial − Final) are given in rows two and three.

## Discussion

Retinal ischemia is typically a temporary condition that can result in permanent vision loss. Under ischemic/hypoxic conditions, irreversible damage to the retina and optic nerve begins to occur at approximately 90 min [1, 33, 34], motivating rapid application of hypothermic therapy following ischemic onset. Using the scleral contact eye cooler (SCEC) evaluated here, all temperature sensor positions reached therapeutically relevant temperatures in less than 12 min. In this *in vitro* model, the optic nerve experienced the least cooling; however, the final temperature reached during active cooling (30.2 °C, Optic Nerve column in Table 3) is close to the 30 °C shown to afford measurable neuroprotection in retinal ganglion cells [23, 24] and recovery of electrophysiological function following retinal ischemia [35]. All other sensor locations reached temperatures below 30 °C, implying that the entire retina achieves therapeutically low temperatures.

One limitation of the present study is the use of a non-perfused eye model. The robust retinal and choroidal blood flows are expected to play a significant role in redistributing heat throughout the eye tissues. In a healthy eye, the long posterior ciliary arteries enter the eye globe on two sides and travel anteriorly to supply the major arterial circle of the iris and anterior choroid (close to the SCR contact area), where the blood would presumably be cooled by the SCEC. The cooled blood would then flow in a posterior direction through anterior ciliary veins to the vortex vein that exits the eye globe posterior to the equator [36]. Blood flow in the posterior part of the eye begins with the short posterior ciliary arteries that enter near the optic nerve; blood then flows anteriorly and exits at the vortex veins just posterior to the equator [36]. This direction of flow would presumably bring heat into the posterior globe and forward, working against the anterior–posterior cooling of the SCEC. The inner retina's blood supply enters at the optic nerve via the central retinal artery, which branches and perfuses the inner layers of the retina, with venous return flowing back toward the posterior pole and exiting the eye at the optic nerve via the central retinal vein, bringing cooled blood toward the back of the eye [36]. The inner retina blood flow is approximately 1/10 that of choroidal blood flow in rats [37], but the capillaries are positioned within the retina itself, which is the target for neuroprotection.

Given the general vascularization schemes described above, normal ocular blood flow could reduce the temperature gradient between anterior and posterior tissues, allowing lower temperatures to be reached at the posterior pole for the same safe SCR temperature compared to the non-perfused model used here. The main indication for therapeutic hypothermia is ischemia, and so a reduced perfusion model would likely be most representative of the clinical application. In addition, other aspects of ocular thermoregulation may influence temperature distribution within the eye, including the role of choroidal thickness changes and flow adjustments in response to thermal stress [38–40]. An *in vitro* perfused eye model or large animal *in vivo* experiments will help clarify the relative contributions of blood flow on temperatures throughout the eye.

Other limitations of the study, likely underlying the observed inter-eye differences in temperature vs*.* time results at each target sensor position (row 4 of Table 2), include potential variability in contact pressure of the SCR on the sclera (potentially affecting thermal transfer between the eye and the device), inconsistency in placement of the implanted sensors, occasional introduction of small air bubbles during the implantation process, and not reaching more uniform temperatures prior to setting the SCR to 4 °C. These issues are being addressed as we refine the system for future experiments. Even with the present limitations, the results were similar across the five eyes.

This study sought to determine if active cooling of the eye using a scleral contact device could produce therapeutically relevant cooling in the posterior ocular tissues. This goal was achieved using an eye similar in size and gross anatomy to adult human eyes. A design goal for the SCEC was to ensure that the eye contact portion is, with some refinement, anatomically amenable to *in vivo* use in human subjects. Effective cooling was achieved with a scleral contact ring (SCR) that is similar in shape to the rigid speculum integral to a commonly used contact lens sensor used in electroretinography (Burian-Allen Electrode, Hansen Ophthalmic Development Laboratory, Bellingham, WA), which is worn for up to an hour during routine clinical testing. Future ECR designs will reduce the bulk of the device to improve comfort while keeping the intended scleral contact area (Fig. 2C) constant.

Validation of effective cooling with this interface will enable critical *in vivo* pre-clinical studies in large animal models, allowing eventual histological analyses of cellular damage (safety) and neuroprotection (efficacy). These pre-clinical studies will directly support clinical trials in human subjects, where SCEC-mediated hypothermia could be used with established clinical tools (e.g., electroretinography) to evaluate retinal health, with the ultimate goal of demonstrating effective neuroprotection following ischemic events. Full clinical translation would likely involve the development of both desk-top (clinic) and portable (field use) versions of the SCEC system to ensure that neuroprotective cooling is available at the earliest possible time to minimize avoidable vision loss.

## Data Availability

Data are available from the authors upon reasonable request.
